# Alterations in Running Biomechanics after 12 Week Gait Retraining with Minimalist Shoes

**DOI:** 10.3390/ijerph17030818

**Published:** 2020-01-28

**Authors:** Yang Yang, Xini Zhang, Zhen Luo, Xi Wang, Dongqiang Ye, Weijie Fu

**Affiliations:** 1 School of Kinesiology, Shanghai University of Sport, Shanghai 200438, China; yangyang.sus@gmail.com (Y.Y.); xinizhang.sus@gmail.com (X.Z.); luozhen716@gmail.com (Z.L.); zwx252@163.com (X.W.); yedongqiang09@gmail.com (D.Y.); 2 Key Laboratory of Exercise and Health Sciences of Ministry of Education, Shanghai University of Sport, Shanghai 200438, China

**Keywords:** gait retraining, running biomechanics, strike pattern, minimalist shoe

## Abstract

*Purpose*: The intervention of 12 week gait retraining with minimalist shoes was established to examine its effect on impact forces, joint mechanics, and vertical stiffness during running. *Methods*: Thirty male recreational runners were randomly assigned to the gait retraining + minimalist shoe (*n* = 15, GR) and minimalist shoe (*n* = 15, MIN) groups. The ground reaction force and marker trajectories were collected before and after intervention at a speed of 3.33 ± 5% m/s. *Results*: A total of 17 participants (9 in the GR group and 8 in the MIN group) completed the training. After training, (1) the loading rate of both groups decreased significantly, and the loading rate of the GR group was lower than that of the MIN group. (2) The foot strike angle of the GR group decreased significantly after training, and the plantarflexion angle and hip joint angular extension velocity increased in both groups. (3) The moment of ankle joint increased in the GR group, and the stiffness of lower limbs was significantly improved in both groups. *Conclusion*: The 12 week gait retraining with minimalist shoes converted rearfoot strikers into forefoot strikers with a rate of 78% (7/9). More importantly, such a combined program, compared to the training with only minimalist shoes, can avoid the peak impact force and decrease the loading rate more effectively, thus providing a potential means of reducing risk of running injury caused by impact forces. Moreover, the increased vertical stiffness of lower extremity after gait retraining may improve running economy and corresponding energy utilization. However, these observations also suggest that the sole use of minimalist footwear may have limited effects on reducing running-related impacts.

## 1. Introduction

As one of the most popular sports in the world, running is attracting increasing attention nowadays [1]. However, a high injury rate (19–79%) in running has been reported [2]. The impact load is two to three times of the body weight at touchdown, which is considered to be the main risk factor for causing damage such as stress fracture/fracture, patellofemoral joint pain syndrome, and plantar fasciitis [2,3,4,5]. Thus, how to reduce the impact and risk of running injury has always been a hot issue in the biomechanics, sports medicine, rehabilitation, and related industries [6,7].

In the past 50 years, the injury rate of running has not changed much despite the development of running shoes [8]. Studies show that the cushioning function of running shoes cannot be utilized in actively landing [6,9,10]. Hence, researchers have considered different shoe designs and the postural control in lower limbs whilst running. As a result, gait retraining and minimalist shoe training derived from barefoot running theory have been applied to rehabilitation, medical treatment, and sports fields [11,12,13,14].

Minimalist footwear are shoes with a lighter mass, greater flexibility, and lower heel-to-toe drop than conventional running shoes [15]. Runners who use this type of footwear likely adopt a non-rearfoot strike pattern [16,17], which can reduce impact forces [11,16]. McCarthy et al. [13] showed that after a 12 week simulated barefoot training, in which the participants were free to adopt their own running pattern, 100% used non-rearfoot strike patterns. Latorre-Roman et al. [18] found that a 12 week barefoot training program causes significant changes in the foot strike pattern, with a tendency towards midfoot or forefoot strikes. However, not all runners who are used to wearing conventional shoes can switch to non-rearfoot strike when wearing minimalist shoes [18]. Without the cushioning of conventional shoes, the risk of high impact-related injuries likely increases [19,20]. Therefore, combining gait retraining and minimalist shoes may be more effective and secure than adopting the two separately.

Gait retraining, an active training program with instruction or feedback, differs from the minimalist shoe training, which is a passive adaptive process of special shoe conditions (e.g., minimalist shoes, barefoot shoes). Promoting a forefoot strike pattern, which is similar to the barefoot movement in the literature, is considered as a possible way forward [17,21]. In addition to promoting non-rearfoot strike patterns, gait retraining encourages forefoot/midfoot strike for a high frequency, light stride and an upright posture [21,22]. Gait retraining reduces loading rate and impact peak by increasing the stride frequency and adopting a non-rearfoot strike pattern [16,21,23]. Warne et al. [12] showed that a 6 week combination program of gait retraining with minimalist shoes causes more significant changes than that of gait retraining with conventional shoes. In light of the above information, the combination of gait retraining and minimalist shoes can reduce the loading rate and peak impact force by using a non-rearfoot strike pattern [13,16,23]. The implication is that the shoe condition should match the running posture. However, the long-term impact on the running posture of such a combination program remains unclear. The combined training program with a long incremental load may be effective and safe.

The purpose of this study was to establish a combined intervention mode of 12 week gait retraining with minimalist shoes and examine its effect on factors related to the risks of running injury and performance, i.e., impact forces, joint mechanics, and vertical stiffness. The hypothesis was that the participants received 12 week gait retraining with minimalist shoes would have a lower loading rate and a decreased foot-strike angle compared to that of those who only used minimalist shoes.

## 2. Methods

### 2.1. Participants

Thirty recreational male runners (age: 30.0 ± 6.4 years; height: 175.0 ± 5.2 cm; body mass: 71.9 ± 9.4 kg; weekly running volume: 27.4 ± 8.7 km) were recruited. Inclusion criteria are as follows: (1) they ran at least 3 days per week with a minimum of 20 km/week for at least 3 months prior to the study and (2) they were used to running with rearfoot strike in cushioned shoes and had no experience of barefoot running or special sneakers (e.g., five-finger shoes, minimalist shoes, and racing spikes). Prior to this experiment, participants completed a basic information questionnaire and signed an informed consent form to ensure that they had no musculoskeletal injuries for the past 6 months. This study was approved by the Institutional Review Board of the Shanghai University of Sport (no. 2017007).

### 2.2. Experimental Design

**Figure 1:**
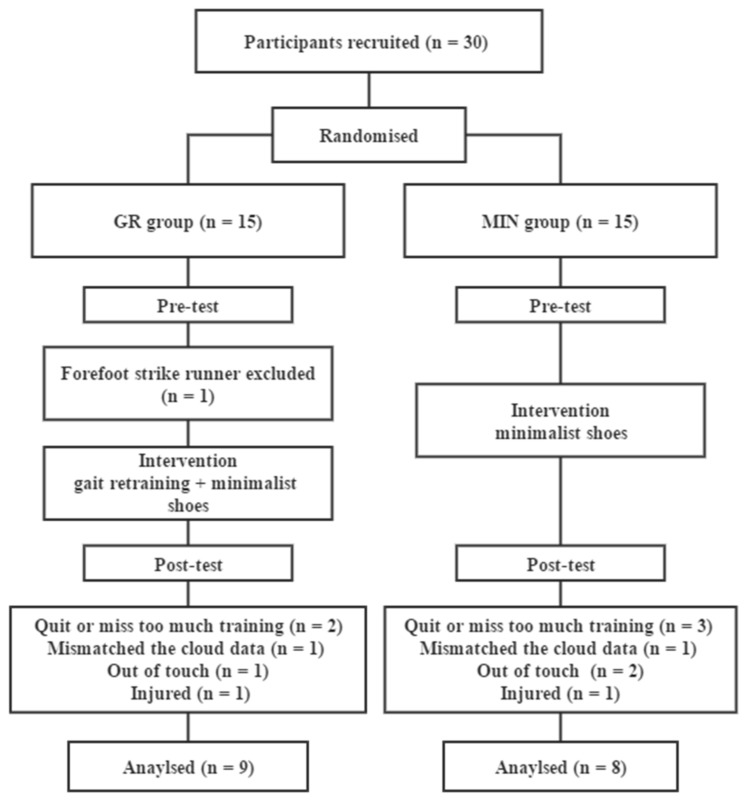
Flow diagram of this study.

A parallel randomized control design was used in this study. Thirty participants were randomly (random number sort) divided into gait retraining + minimalist shoe (GR) and minimalist shoe (MIN) groups. The two groups underwent the same testing process but with different interventions (Figure 1). Foot size was measured, and participants in both groups were provided with a pair of minimalist footwear (type INOV-8 Bare-XF 210 V2: 3 mm outsole, no midsole, 0 mm heel-toe drop, 227 g weight).

### 2.3. Testing Procedure

**Figure 2:**
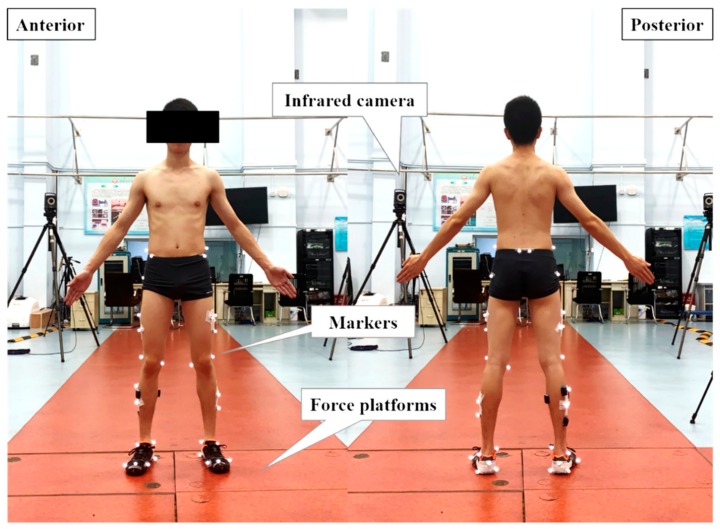
The marker-set and the experimental setup.

A 10-camera motion capture system (100 Hz, T40, Vicon Motion Inc., Oxford, United Kingdom) was used to collect kinematic data including hip, knee, and ankle joints (Figure 2). Two 90 × 60 × 10 cm Kistler 3D force platforms (9287B, Kistler Corporation, Winterthur, Switzerland) were used to collect ground reaction force (GRF) data at a sampling rate of 1000 Hz. Before the over-ground test, the participants performed a 5 min warm-up on a treadmill at optional running speed with the minimalist shoes, followed by 1 min 3.33 m/s experimental speed adaptation. During the over-ground test, three successful right foot contacts on the force plate were required. Its presence was not mentioned to avoid targeting problems. The speed during the over ground test was monitored to ensure the participants ran at 3.33 m/s using a Witty-Manual grating timing system (Witty wireless training timer, Microgate Corp., Bolzano, Italy) with a 5% acceptable variance. Both the GR and the MIN groups were tested before and after the intervention.

### 2.4. Intervention

GR group: The participants were required to run at a medium-intensity self-selected speed with minimalist shoes and strike with forefoot. A pressure sensitive insole (Podoon) was applied to GR runners to distinguish foot strike patterns. The sensors were located at the metatarsophalangeal joint and heel. Sound feedback could be obtained from a mobile application if participants struck with the heel. The gait retraining program lasted 12 weeks and was three times a week. The duration of the training gradually increased from 5 min in the 1st week to 48 min in the 12th week (Table 1) [12,13]. Weekly group training was also provided to ensure the quality of retraining and to minimize the dropout rates. After each training, the experimenter will remind the participants who do not meet the requirements or mismatch with the data in the cloud.

MIN group: During the running training, the participants were required to run at a medium-intensity self-selected speed wearing minimalist shoes without any instructions for the strike pattern. A pressure-sensitive insole (Podoon) was also applied to MIN runners for matching the same insole condition of the GR group, but they did not receive the mobile application that provided sound feedback. The schedule was the same as that of the GR group.

The intervention training was only an alternative part of the training [12,13]. The total running distance per week was unchanged. Participants kept record training logs, including the time training start/stop, location, and distance. During training, they were told that any discomfort or injury needed to be reported to the experimenter. The researchers checked the training logs stored in the cloud.

The participants in both groups were allowed to wear habitual running shoes when out of training. During training sessions, the two groups were prevented from interacting with one another.

Inclusion criteria: (1) completed all tests, (2) no more than three absences, and (3) completed the last 3 weeks’ training with no more than six absences. Those who satisfied any condition were included. During training, participants were allowed to delay or withdraw due to injury or personal reasons.

### 2.5. Data Processing

Kinematic data and GRF were analyzed via the gait analysis software Visual 3D (v5, C-Motion, Inc., Germantown, MD, USA) using inverse dynamics. The GRF was filtered with a cut-off frequency of 100 Hz. Marker trajectories were filtered with a cut-off frequency of 7 Hz [10] via a fourth-order Butterworth low-pass filter. The hip, knee, and ankle angles of the lower limb were defined on the basis of our previous model [24], and the kinematic features of each joint were calculated.

Impact variables included peak impact forces and maximum loading rates. The maximum loading rate (LR) is equivalent to a slope of 20–80% of first peak (FP). If FP is non-existent, then LR is calculated by using 13% of the gait cycle as a representative value [25,26].

**Figure 3:**
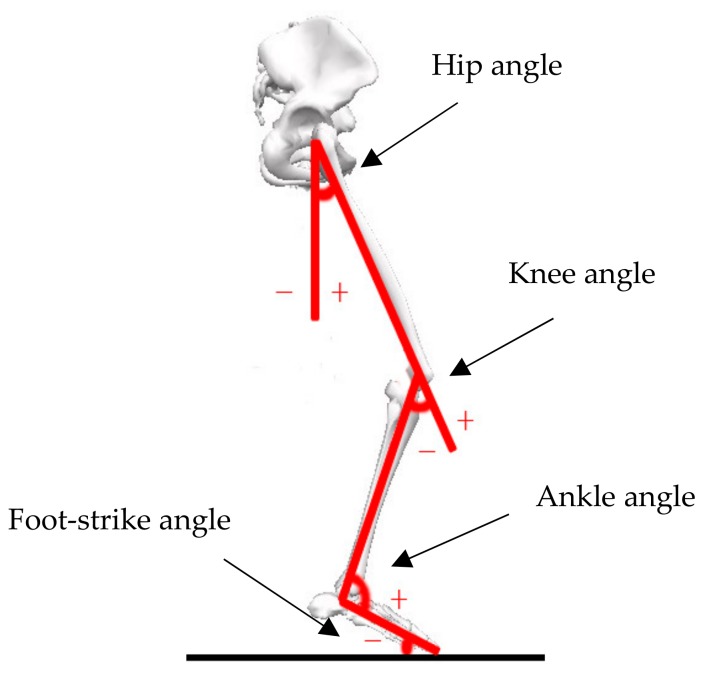
Angles of lower extremity joints.

Kinematic variables included (1) ground contact time (CT), which represents the duration between touchdown to off-ground; (2) strike angle (*θ_f_*) which refers to the angle between the foot and ground at initial contact; (3) angles of the hip, knee, and ankle joints when contacting the ground (*θ_0_*) and the maximum joint angle (*θ_max_*); and (4) joint angular velocities including the angular velocity at initial contact (*ω_0_*) and the maximum angular velocity of hip, knee, and ankle joints (*ω_p_*). The angle of ankle joint was 0° during standing, negative for extension/plantarflexion, and positive for flexion/dorsiflexion (Figure 3).

Kinetic variables included (1) joint moment determined by the net moment generated by the muscles of the hips, knees, and ankles of the lower limbs using the inverse dynamics in Visual 3D biomechanical analysis software; (2) peak extension joint power (*p*), which is the product of the net moment (*M*) and joint angular velocities (*ω*), and (3) vertical stiffness (*k* = GRF*_i_*/Δ*y*) [27]. For the joint moment, the maximum extension moment (*M*_max_) of each joint was selected. GRF*_i_* represents the vertical GRF when the center of gravity (CoG) was lowest, and Δ*y* represents the vertical displacement of CoG during centrifugation.

### 2.6. Statistics

The mean and standard deviation for each variable were calculated. The results of each group of pre/post were tested for normality. The original value was used in all tables and figures for comparison. A two-way repeated measure ANOVA was used to examine the effects of retraining (pre- and post-training) and groups (GR and MIN) on each variable (Version 22.0; SPSS, Inc., Chicago, IL, USA). Independent *t*-tests and paired *t*-tests were used as post-hoc tests when a significant interaction was detected. The significance level was set as α = 0.05.

## 3. Results

### 3.1. Dropout Rate

Seventeen participants completed intervention and met the inclusion criteria (nine in the GR group, eight in the MIN group) (Table 2). Specifically, an FFS runner in the GR group was excluded after pre-test. During intervention, two participants (one in GR, one in MIN) were excluded due to injuries caused by non-training related events, i.e., walked downstairs carelessly. Two participants (one in GR, one in MIN) were excluded due to mismatch of the cloud data, and they could not provide reliable evidence, such as app or smart watch data. Three participants (one in GR, two in MIN) who lost contact during the training were excluded. Five participants (two in GR, three in MIN) who quit or missed too much training were also excluded. No significant difference was observed in the average running volumes between the GR and MIN groups (GR: 28.3 ± 11.2 km/week, MIN: 26.9 ± 10.7 km/week).

### 3.2. Impact Forces

**Figure 4:**
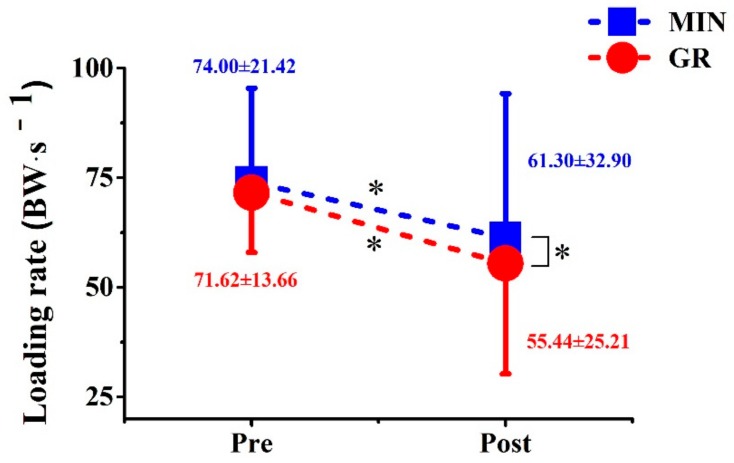
Comparison of loading rate between two groups before and after training.

A significantly main effect of time on the loading rate was observed (Figure 4; Table 3), which was significantly reduced by 22.6% (GR) and 17.2% (MIN) after training (*p* < 0.001, *p* = 0.017). The loading rate of the GR group was lower than that of the MIN group after training (*p* = 0.015). No interaction effect was noted between time × group for any other GRF parameters in this study.

### 3.3. Kinematics

**Figure 5:**
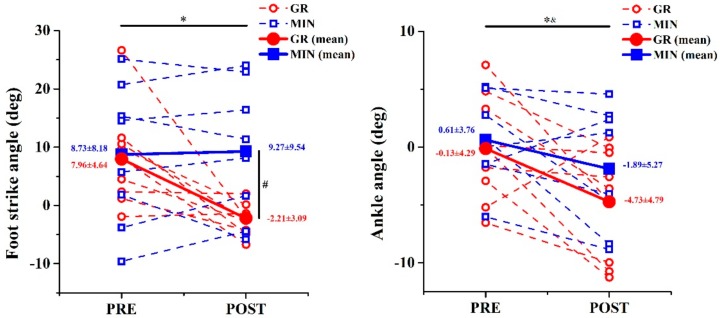
Comparison of foot-strike angle (left) and ankle angle (right) between two groups before and after training. * significant difference from pre- to post-tests in GR group; ^#^: significant difference between groups at time point, *p* < 0.005; &: significant difference from pre- to post-tests in MIN group.

A significant main effect of time was observed on the foot-strike angle, ankle angle (Figure 5), and angular velocity of hip (*p* = 0.026, *p* = 0.011, *p* = 0.032, respectively) (Table 4). In addition, a significant interaction effect between time × group on the foot-strike angle was observed (*p* = 0.013). After the post-hoc test, the foot-strike angle of the GR group decreased by 10.3° after training (*p* = 0.015), but no difference was noted in the MIN group (*p* = 0.753) (Figure 5). The foot-strike angle of the GR group was significantly different from that of the MIN group in the post-test (*p* = 0.017). After training, the ankle angle significantly decreased by 4.6° (GR) and 2.5° (MIN) at touchdown, and the maximum angular velocity of hip joint increased by 15.2% (GR) and 25.2% (MIN). There was no interaction effect between time × group for any other kinematic parameters.

### 3.4. Kinetics

**Figure 6:**
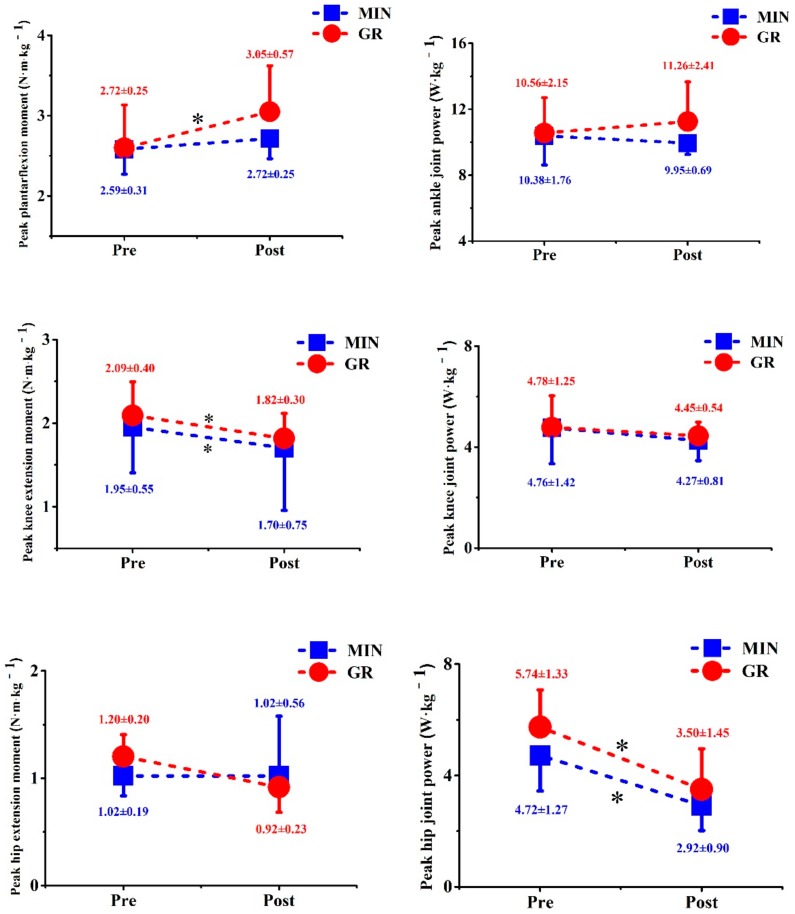
Comparison of peak moment (left column) and peak power (right column) of hip, knee, and ankle between two groups before and after training. * significant difference from pre- to post-tests.

A significant main effect was observed on the peak ankle extension moment (*p* < 0.001), peak knee extension moment (*p* = 0.004), and peak power of the hip (*p* < 0.001) (Figure 6). The peak moment of the knee for the GR and MIN groups significantly decreased by 13.4% (GR) and 12.8% (MIN), respectively, after training, and the peak power of the hip for both groups was significantly decreased by 38.6% (GR) and 38.2% (MIN). In addition, a significant interaction effect was noted on the peak moment of ankle (*p* = 0.024). Specifically, the peak moment was increased by 17.8% after training in the GR group (*p* = 0.001), but no difference was observed in the MIN group.

### 3.5. Vertical Stiffness

**Figure 7:**
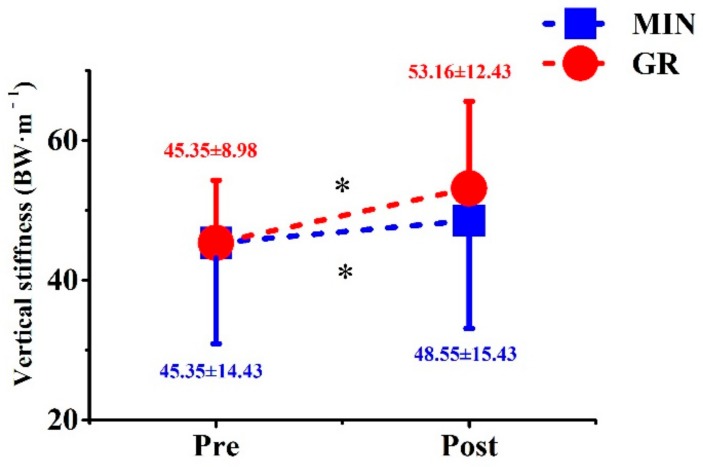
Comparison of lower limbs stiffness between two groups before and after training. * significant difference from pre- to post-tests.

A significant main effect of time was observed on vertical stiffness (*p* = 0.035). After training, the vertical stiffness improved in the GR and MIN groups by 17.2% and 7.1%, respectively (Figure 7). However, no significant main effect or interaction was observed on the vertical displacement of CoG and the vertical GRF (Table 5).

## 4. Discussion

The purpose of this study was to examine the effect of gait retraining with minimalist shoes on impact forces, joint mechanics, and vertical stiffness. Significant reduction was found in foot-strike angle and LR. The kinematics and kinetics of the lower extremity joints changed after 12 weeks of gait retraining with the minimalist shoes. Compared with the MIN group, more significant changes were noted in the GR group, especially in the strike patterns and kinematics and kinetics characteristics of the ankle joint. These results supported the hypothesis that the participants who received 12 week gait retraining with minimalist shoes would have a lower loading rate and more lower foot strike angle compared to that of those who only used minimalist shoes.

### 4.1. Impact Forces

Gait retraining can significantly reduce the impact force, which is considered to be the main cause of lower-limb injuries, and this result supports the findings of previous studies [2,3,4]. Seven out of nine participants of the GR group changed to forefoot strike after gait retraining. Previous studies demonstrate that the impact force mainly depends on the effective mass of the lower limbs [11]. The forefoot strike can obviously reduce the effective mass by adjusting the angle between the foot and the ground at initial contact, thus avoiding the high impact force caused by rearfoot strike.

The LR, the change in force per unit time, is considered to be a sensitive index to detect the variations in the amount of impact forces during running. In this study, the LR for both groups decreased after training. For the GR group, the change of the strike pattern avoided the peak of impact force, thereby reducing the LR. This outcome is similar to the findings of other related studies [2,3,4,12,25,26]. By contrast, the MIN showed a significantly reduced LR without the change of strike pattern, which may be related to the adaptability of the body after the change of shoe condition [11]. The LR of the GR group was lower than that of the MIN group after training, indicating that the effect of gait retraining may be more significant. Gait retraining as an intervention for actively changing the strike pattern may be better matched with minimalist shoes to reduce the LR and avoid the peak of the impact force.

### 4.2. Kinematics

The participants in the GR group preferred to strike with the forefoot after training, as shown by the foot-strike angle reduced by approximately 10.3°. This preference of forefoot strike in the GR group was the embodiment of the gait retraining [11]. After 12 weeks of training, the participants in the GR group ran similarly to runners who run barefoot.

In the MIN group, no change was noted in strike pattern after training. In the study of McCarthy et al. [13], all participants who preferred rearfoot strike became non-rearfoot strike after training with minimalist shoes. Latorre-Roman et al. [18] revealed that a 12 week barefoot running program causes significant changes in foot strike patterns with a tendency towards a non-rearfoot strike in long-distance runners. In the study of Hollander et al. [28], habitually shod participants who actively changed from shod to barefoot increased the foot strike index. However, the results in the current study did not show this result, which might have been due to the difference in training program or the gender, age, and other attributes of the participants.

Although only runners in the GR group showed a significant change in foot-strike pattern, the two groups exhibited more plantarflexion after training. For the GR group, the larger plantarflexion angle indicated a trend towards forefoot strike pattern [11,29]. In this study, the variation of the plantarflexion angle of the GR group was not larger than the foot-strike angle (10.3° vs. 4.8°). This outcome indicates that the runners in GR may not achieve forefoot strike by simply increasing the plantarflexion angle. No significant change was observed in knee and hip angles when touching the ground. Apart from increasing the plantarflexion angle of ankle, the participants in the GR group might have achieved the forefoot strike pattern by adjusting the position of the body and the forward of the trunk after training instead of simply “running on the toe” [11].

### 4.3. Kinetics and Vertical Stiffness

The peak ankle moment increased significantly in the GR group after training, but no significant difference was observed in the MIN group. This outcome may have been due to increasing arm of force changed by the foot strike pattern. Warne et al. reported no significant change in the stiffness of ankle after gait retraining [12]. Although the peak ankle moment increased, runners maintained the stiffness by increasing the range of ankle upon touchdown. In the current study, this phenomenon may have appeared as the increased plantarflexion angle. The peak knee extension moment for both groups decreased significantly after training. When wearing minimalist shoes, the reduced stiffness of the knee may result in reduced moments or increased knee excursion compared with wearing traditional shoes [12], suggesting that the knee is more inclined to soft landing in the case of minimalist shoes.

In this study, the peak power of hip of both groups decreased significantly. In the study by Williams et al. [30], runners wearing shoes showed a significant decrease in the power of hip when using forefoot strike pattern or barefoot running compared with rearfoot strike pattern, and this effect continued after training. Similarly, in the present study, the vertical stiffness, which is considered to be related to running economy and energy utilization, increased significantly after training in both groups [31]. Gait retraining or using minimalist shoes may improve running performance. The results also showed that the vertical GRF and the displacement of CoG did not change significantly after training. Hence, vertical stiffness seems better for evaluating the effectiveness of the training.

During the intervention, two participants were injured due to non-training factors and they were excluded from the statistics of the proportion of injuries. In other related studies, the proportion of injuries in McCarthy’s study was 20–26% [13], and the proportion was 17% for two runners who were injured in the study of Warne et al. [12]. The number of injured in the present study was also two. This outcome suggests that gait retraining is important when running barefoot or wearing minimalist shoes.

### 4.4. Limitations

Firstly, the sample size was relatively small due to the long training period and participant dropout. A large sample size could have increased the statistical power in such a way that additional variables achieved significance, especially the variable trends in kinematics but without significance. For future research on recreational runners, additional attention should be paid to the training control of the participants due to work travel and other reasons, which are difficult but useful for the sample preservation. In addition, this study focuses on the contrast between two different training programs (GR and MIN). Thus, no barefoot and control groups are set up. However, a complete four-group study (combined, control, minimalist, and barefoot groups) is necessary for future research. Secondly, individual differences in movement learning ability might have led to different training effects, especially in the GR group. Moreover, the long-term retention effects caused by retraining changes are unknown. Finally, future investigations, including EMG assessment accompanied with neuro-musculoskeletal adaptations after gait retraining, are warranted.

## 5. Conclusions

The 12 week gait retraining with minimalist shoes converted rearfoot strikers into forefoot strikers. Seven of out nine participants transformed into forefoot strike patterns with a rate of 78%. More importantly, such a combined program, compared to the training with only minimalist shoes, can avoid the peak impact force and decrease the loading rate more effectively, thus providing a potential means of reducing risk of running injury caused by impact forces. Moreover, the increased vertical stiffness of lower extremity after gait retraining may improve running economy and corresponding energy utilization. However, these observations also suggest that the sole use of minimalist footwear may have limited effects on reducing running-related impacts.

## Figures and Tables

**Table 1:** The 12 week gait retraining intervention.

Week	1	2	3	4	5	6	7	8	9	10	11	12
Duration (min)	5	10	15	20	25	30	35	40	42	44	46	48
Times per week	3	3	3	3	3	3	3	3	3	3	3	3

**Table 2:** Information of the participants who completed training. GR: gait retraining + minimalist shoe, MIN: minimalist shoe.

	Age (years)	Height (cm)	Body Mass (kg)	km per Week (km)
GR (*n* = 9)	32.4 ± 6.1	174.8 ± 5.3	70.2 ± 6.0	28.3 ± 11.2
MIN (*n* = 8)	27.6 ± 5.2	173.9 ± 7.0	75.4 ± 11.7	26.9 ± 10.7
*t*-test	*p* = 0.104	*p* = 0.773	*p* = 0.262	*p* = 0.787

**Table 3:** Contrast of ground reaction force (GRF) in different shoe conditions before and after training.

Parameter	GR		MIN	
	Pre	Post	Pre	Post
FP (BW)	1.78 ± 0.20	N/A	1.83 ± 0.22	2.05 ± 0.47
T_FP_ (ms)	25.50 ± 5.31	N/A	26.20 ± 4.71	28.97 ± 15.75
LR (BW·s^−1^)	71.62 ± 13.66 ^#^	55.44 ± 25.21 *	74.00 ± 21.42	61.30 ± 32.90
CT (ms)	233.58 ± 20.44	226.35 ± 11.90	243.27 ± 26.65	240.02 ± 26.26

FP: the first peak of the touch-down phase; T_FP_: the instant reaching the FP; BW: body weight; LR: loading rate; CT: ground contact time; ^#^: significant difference from pre- to post-tests. * significant difference between groups at time point, *p* < 0.005.

**Table 4:** Kinematics changes of hip, knee, and ankle before and after training.

Joints	Parameter	GR		MIN	
		Pre	Post	Pre	Post
Foot /Ankle	*θ_f_* (deg)	8.07 ± 4.64 *^,&^	−2.21 ± 3.09 ^#^	8.73 ± 6.68	9.27 ± 8.9
	*θ_0_* (deg)	−0.13 ± 4.29 *	−4.73 ± 4.79 ^#^	0.61 ± 3.76 *	−1.89 ± 5.27
	*θ_max_* (deg)	17.48 ± 4.76	16.92 ± 4.78	15.91 ± 2.51	16.58 ± 3.38
	*ω*_0_ (deg·s^−1^)	289.57 ± 85.81	332.77 ± 103.51	259.22 ± 34.13	267.83 ± 67.03
	*ω_p_* (deg·s^−1^)	−269.34 ± 90.64	−245.00 ± 60.65	−231.46 ± 66.38	−213.37 ± 44.05
Knee	*θ_0_* (deg)	−13.56 ± 5.60	−14.24 ± 5.11	−14.17 ± 4.01	−11.80 ± 3.76
	*θ_max_* (deg)	−34.46 ± 2.08	−35.41 ± 4.75	−35.47 ± 2.93	−36.15 ± 4.62
	*ω*_0_ (deg·s^−1^)	−96.83 ± 35.33	−85.96 ± 51.95	−85.80 ± 39.66	−83.61 ± 57.09
	*ω_p_* (deg·s^−1^)	103.52 ± 34.96	109.21 ± 26.37	90.68 ± 40.83	74.21 ± 22.57
Hip	*θ_0_* (deg)	25.50 ± 5.04	27.36 ± 8.23	29.36 ± 5.36	28.25 ± 7.28
	*θ_max_* (deg)	−11.06 ± 6.59	−10.74 ± 7.30	−7.92 ± 6.03	−9.73 ± 6.71
	*ω*_0_ (deg·s^−1^)	−82.08 ± 29.88	−60.34 ± 15.15	−64.37 ± 43.64	−62.22 ± 21.88
	*ω_p_* (deg·s^−1^)	95.49 ± 39.13 *	109.99 ± 26.54	100.45 ± 26.82 *	125.73 ± 28.51

*θ_f_*: the angle between foot and ground at initial contact; *θ*_0_: the angle at initial contact; *θ*_max_: maximum angle; *ω*_0_: the peak angular velocity at initial contact; *ω*_p_: the peak angular velocity of extension; * significant difference from pre- to post-tests; ^#^ significant difference between groups at time point, *p* < 0.005; &: interaction effect between time × group, *p* < 0.005.

**Table 5:** Vertical GRF when the center of gravity (CoG) was lowest, and the vertical displacement of CoG. GRFi represents the vertical GRF when the CoG was lowest, and Δy represents the vertical displacement of CoG during centrifugation.

Parameter	GR		MIN	
	Pre	Post	Pre	Post
GRF*i* (BW)	2.61 ± 0.30	2.71 ± 0.31	2.55 ± 0.28	2.60 ± 0.27
Δ*y* (cm)	5.96 ± 0.90	5.42 ± 0.99	6.21 ± 1.43	5.67 ± 1.47

## References

[1] Bramble D.M., Lieberman D.E. (2004). Endurance running and the evolution of Homo. Nature.

[2] Van Gent R.N., Siem D., van Middelkoop M., van Os A.G., Bierma-Zeinstra S.M., Koes B.W. (2007). Incidence and determinants of lower extremity running injuries in long distance runners: A systematic review. Br. J. Sports Med..

[3] Milner C.E., Ferber R., Pollard C.D., Hamill J., Davis I.S. (2006). Biomechanical factors associated with tibial stress fracture in female runners. Med. Sci. Sports Exerc..

[4] Pohl M.B., Hamill J., Davis I.S. (2009). Biomechanical and anatomic factors associated with a history of plantar fasciitis in female runners. Clin. J. Sport. Med..

[5] Wang H., Kia M., Dickin D.C. (2019). Influences of load carriage and physical activity history on tibia bone strain. J. Sport Health Sci..

[6] Fu W., Liu Y., Zhang S. (2013). Effects of footwear on impact forces and soft tissue vibrations during drop jumps and unanticipated drop landings. Int. J. Sports Med..

[7] Fu W., Wang X., Liu Y. (2015). Impact-induced soft-tissue vibrations associate with muscle activation in human landing movements: An accelerometry and EMG evaluation. Technol. Health Care.

[8] Nigg B.M. (2001). The role of impact forces and foot pronation: A new paradigm. Clin. J. Sport. Med..

[9] Wang X., Zhang S., Fu W. (2017). Changes in Impact Signals and Muscle Activity in Response to Different Shoe and Landing Conditions. J. Hum. Kinet..

[10] Fu W., Fang Y., Gu Y., Huang L., Liu Y. (2017). Shoe Cushioning Reduces Impact and Muscle Activation during Landings from Unexpected, but not Self-initiated, Drops. J. Sci. Med. Sport.

[11] Lieberman D.E., Venkadesan M., Werbel W.A., Daoud A.I., D’Andrea S., Davis I.S., Mang’eni R.O., Pitsiladis Y. (2010). Foot strike patterns and collision forces in habitually barefoot versus shod runners. Nature.

[12] Warne J.P., Smyth B.P., Fagan J.O., Hone M.E., Richter C., Nevill A.M., Moran K.A., Warrington G.D. (2017). Kinetic changes during a six-week minimal footwear and gait-retraining intervention in runners. J. Sports Sci..

[13] McCarthy C., Fleming N., Donne B., Blanksby B. (2014). 12 weeks of simulated barefoot running changes foot-strike patterns in female runners. Int. J. Sports Med..

[14] Daoud A.I., Geissler G.J., Wang F., Saretsky J., Daoud Y.A., Lieberman D.E. (2012). Foot strike and injury rates in endurance runners: A retrospective study. Med. Sci. Sports Exerc..

[15] Lussiana T., Hébert-Losier K., Mourot L. (2015). Effect of minimal shoes and slope on vertical and leg stiffness during running. J. Sport Health Sci..

[16] Altman A.R., Davis I.S. (2012). A kinematic method for footstrike pattern detection in barefoot and shod runners. Gait Posture.

[17] Giandolini M., Arnal P.J., Millet G.Y., Peyrot N., Samozino P., Dubois B., Morin J.-B.t. (2013). Impact reduction during running: Efficiency of simple acute interventions in recreational runners. Eur. J. Appl. Physiol..

[18] Latorre-Roman P.A., Garcia-Pinillos F., Soto-Hermoso V.M., Munoz-Jimenez M. (2019). Effects of 12 weeks of barefoot running on foot strike patterns, inversion-eversion and foot rotation in long-distance runners. J. Sport Health Sci..

[19] Ryan M., Elashi M., Newsham-West R., Taunton J. (2014). Examining injury risk and pain perception in runners using minimalist footwear. Br. J. Sports Med..

[20] Salzler M.J., Bluman E.M., Noonan S., Chiodo C.P., de Asla R.J. (2012). Injuries Observed in Minimalist Runners. Foot Ankle Int..

[21] Goss D.L., Gross M.T. (2012). A review of mechanics and injury trends among various running styles. US Army Med. Dep. J..

[22] Dallam G.M., Wilber R.L., Jadelis K., Fletcher G., Romanov N. (2005). Effect of a global alteration of running technique on kinematics and economy. J. Sports Sci..

[23] Crowell H.P., Davis I.S. (2011). Gait retraining to reduce lower extremity loading in runners. Clin. Biomech..

[24] Xia R., Zhang X., Wang X., Sun X., Fu W. (2017). Effects of Two Fatigue Protocols on Impact Forces and Lower Extremity Kinematics during Drop Landings: Implications for Noncontact Anterior Cruciate Ligament Injury. J. Healthc. Eng..

[25] Blackmore T., Willy R.W., Creaby M.W. (2016). The high frequency component of the vertical ground reaction force is a valid surrogate measure of the impact peak. J. Biomech..

[26] Samaan C.D., Rainbow M.J., Davis I.S. (2014). Reduction in ground reaction force variables with instructed barefoot running. J. Sport Health Sci..

[27] Liu Y., Peng C.H., Wei S.H., Chi J.C., Tsai F.R., Chen J.Y. (2006). Active leg stiffness and energy stored in the muscles during maximal counter movement jump in the aged. J. Electromyogr. Kinesiol..

[28] Hollander K., Liebl D., Meining S., Mattes K., Willwacher S., Zech A. (2019). Adaptation of Running Biomechanics to Repeated Barefoot Running: A Randomized Controlled Study. Am. J. Sports Med..

[29] Bonacci J., Saunders P.U., Hicks A., Rantalainen T., Vicenzino B.G., Spratford W. (2013). Running in a minimalist and lightweight shoe is not the same as running barefoot: A biomechanical study. Br. J. Sports Med..

[30] Williams D.S.B., Green D.H., Wurzinger B. (2012). Changes in lower extremity movement and power absorption during forefoot striking and barefoot running. Int. J. Sports Phys. Ther..

[31] Perl D.P., Daoud A.I., Lieberman D.E. (2012). Effects of footwear and strike type on running economy. Med. Sci. Sports Exerc..

